# Macrophage Depletion Impairs Corneal Wound Healing after Autologous Transplantation in Mice

**DOI:** 10.1371/journal.pone.0061799

**Published:** 2013-04-16

**Authors:** Suxia Li, Bin Li, Haoran Jiang, Yao Wang, Mingli Qu, Haoyun Duan, Qingjun Zhou, Weiyun Shi

**Affiliations:** 1 Qingdao University, Qingdao, China; 2 Shandong Provincial Key Laboratory of Ophthalmology, Shandong Eye Institute, Shandong Academy of Medical Sciences, Qingdao, China; Wayne State University, United States of America

## Abstract

**Purpose:**

Macrophages have been shown to play a critical role in the wound healing process. In the present study, the role of macrophages in wound healing after autologous corneal transplantation was investigated by depleting local infiltrated macrophages.

**Methods:**

Autologous corneal transplantation model was used to induce wound repair in Balb/c mice. Macrophages were depleted by sub-conjunctival injections of clodronate-containing liposomes (Cl_2_MDP-LIP). The presence of CD11b^+^ F4/80^+^ macrophages, α-smooth muscle actin^+^ (α-SMA^+^) myofibroblasts, CD31^+^ vascular endothelial cells and NG_2_^+^ pericytes was examined by immunohistochemical and corneal whole-mount staining 14 days after penetrating keratoplasty. Peritoneal macrophages were isolated from Balb/c mice and transfused into conjunctiva to examine the recovery role of macrophages depletion on wound healing after autologous corneal transplantation.

**Results:**

Sub-conjunctival Cl_2_MDP-LIP injection significantly depleted the corneal resident phagocytes and infiltrated macrophages into corneal stroma. Compared with the mice injected with PBS-liposome, the Cl_2_MDP-LIP-injected mice showed few inflammatory cells, irregularly distributed extracellular matrix, ingrowth of corneal epithelium into stroma, and even the detachment of donor cornea from recipient. Moreover, the number of macrophages, myofibroblasts, endothelial cells and pericytes was also decreased in the junction area between the donor and recipient cornea in macrophage-depleted mice. Peritoneal macrophages transfusion recovered the defect of corneal wound healing caused by macrophages depletion.

**Conclusions:**

Macrophage depletion significantly impairs wound healing after autologous corneal transplantation through at least partially impacting on angiogenesis and wound closure.

## Introduction

Wounds normally heal in an orderly manner through the inflammatory phase, proliferation phase and remodeling phase, which are overlapping and involve many cell types, such as neutrophils, macrophages, fibroblasts and endothelial cells [1]. Infiltrated monocytes and macrophages are key cells in the inflammatory phase and clean the wound bed through the phagocytosis of wound debris and necrotic cells. Moreover, they generate free radicals and produce proinflammatory cytokines to recruit more inflammatory cells to the wound site. In addition, the soluble mediators released are involved in the recruitment and activation of fibroblasts and endothelial cells in preparation for the subsequent proliferative and remodeling phase. Fibroblasts are one of the major cells in the proliferative and remodeling phases, and they migrate and accumulate in the provisional matrix of the wound site and secrete and deposit collagen, proteoglycans and other components of the extracellular matrix (ECM) for tissue matrix remodeling. Moreover, activated fibroblasts can differentiate into myofibroblasts, which are characterized by the expression of the putative contractile marker α-SMA and are central for wound closure. The interactions between myofibroblasts and the surrounding extracellular matrix may be critical for the final result of wound healing. Inappropriate ECM synthesis and deposition can cause fibrosis and scar formation, which results in the deformation of surrounding parenchymal cells [1]–[4].

The cornea, a highly organized avascular tissue located in the anterior part of the eye, has been widely used as a model for the investigation of wound healing [5]–[7]. During corneal wound healing, inflammatory cells infiltrate into the corneal stroma, and quiescent corneal keratocytes are activated and differentiated into fibroblasts and myofibroblasts to repair damaged tissue architecture [8]. In addition, new vessels gradually grow from the limbal region to the central cornea, known as corneal neovascularization. Delayed wound healing often results in chronic inflammation, recurrent epithelial defects, neovascularization and corneal ulceration [9].

The role of macrophages in corneal wound healing has been described in several corneal injury models, such as alkali burn and allogeneic corneal transplantation [10]–[12]. However, the specific role of macrophages in wound healing after autologous corneal transplantation is poorly understood. According to the known function of macrophages in wound healing, we hypothesize that macrophages may play key roles in corneal wound healing after autologous corneal transplantation, and the depletion of infiltrated macrophages into the cornea may impair the repair process. In the present study, we used the liposome-mediated macrophage depletion method to deplete infiltrated phagocytes into the cornea, evaluated the effect of macrophage depletion on the impairment of corneal wound healing after penetrating keratoplasty, including wound closure and angiogenesis.

## Materials and Methods

### Animals

Sixty 6- to 8-week-old Balb/c mice (Beijing Pharmacology Institute, Chinese Academy of Medical Sciences) were used for the experiments. All animal experiments were carried out in accordance with The Chinese Ministry of Science and Technology Guidelines on the Humane Treatment of Laboratory Animals (vGKFCZ-2006-398) and the Association for Research in Vision and Ophthalmology (ARVO) Statement for the Use of Animals in Ophthalmic and Vision Research. The protocol was approved by the Animal Care and Use Committee on the Ethics of the Shandong Eye Institute (Permit Number: SEIRB-2011-81170815). All surgery was performed on only one eye of each mouse under sodium pentobarbital anesthesia, and the mice were sacrificed with an overdose of 10% chloral hydrate.

### Macrophage depletion

Liposomes encapsulated with dichloromethylene diphosphonate (Cl_2_MDP; Sigma, St. Louis, MO) or phosphate-buffered saline (PBS) were prepared and injected as previously described [13], [14]. The final concentration of Cl_2_MDP was 2.5 mg/ml suspension. Mice were injected sub-conjunctivally with 10 µl Cl_2_MDP-LIP (n = 30) under anesthesia on days 7 and 2 before penetrating keratoplasty. Control mice were pretreated with PBS-LIP (n = 30). After phagocytosis and disruption of the phospholipid bilayers, Cl_2_MDP is released into the macrophage cytoplasm, which leads to apoptosis. The depletion of infiltrated macrophages in corneal stroma was evaluated by immunohistochemistry as described below.

### Macrophage transfusion

Peritoneal macrophages were collected by lavage of the peritoneal cavity of naïve Balb/c mice as previously described [15]. As briefly, the fluid from peritoneal cavity after the injection of ice-cold phosphate-buffered saline was centrifuged, and the cell pellet was suspended in RPMI-1640 supplemented with 10% fetal bovine serum. Macrophages were collected by culturing the peritoneal exudate cells for 2 hours at 37°C followed by the removal of non-adherent cells. For the transfusion of macrophages, 10^5^ cells were injected sub-conjunctivally after 48 hours of penetrating keratoplasty.

### Autologous corneal transplantation

Mouse autologous corneal transplantation was performed as described previously, with some modifications [16], [17]. Briefly, 1% atropine eye oil was applied 1 day and 0.5% tropicamide mixed with 0.5% phenylephrine hydrochloride eye drops was used 2–3 times/hour before surgery to dilate the pupil. A 2-mm trephine was used to score the donor cornea before the removal of the corneal button using Vannas scissors. The autologous corneal button was sewn in place using 8 interrupted sutures with 11-0 nylon sutures (Mani, Japan). The anterior chamber was reformed with sterile air. To prevent self-injury to eyes, temporary tarsorrhaphy was performed for 3 days. A topical antibiotic (ofloxacin, Santen, Japan) was applied immediately after surgery and once a day thereafter. The eyes that underwent surgery were monitored at 3, 7, 14, 21 and 28 days, the sutures were removed 10 days after penetrating keratoplasty.

### Histology

Eyes were collected for histology after 14, 21 and 28 days of corneal surgery. The samples were fixed in 4% formalin and embedded in paraffin. Continuous 4 µm sections were stained with hematoxylin & eosin (H.E. staining). The presence of vascular vessels and inflammatory cells and the corneal fiber distribution in the junction between the donor and recipient cornea were observed by light microscopy.

### Immunofluorescence and whole-mount staining

Cornea whole-mount and immunofluorescence staining was performed as previously described [18], [19]. Briefly, full-thickness corneal flat mounts and frozen sections were fixed in acetone for immunofluorescence staining. To block nonspecific staining, the samples were incubated with normal goat serum for 20 minutes at room temperature and incubated with primary antibodies overnight at 4°C. Subsequently, they were incubated with secondary antibodies for 1 hour at room temperature. Each step was followed by 3 wash steps with 0.02 M PBS for 5 minutes. The following primary antibodies were used in the present study: rat anti-F4/80 (MCA497GA, AbD Serotec, Oxford, UK), anti-CD11b (101201, BioLegend, San Diego, CA), anti-α-smooth muscle actin (ab5694, Abcam, Cambridge, MA), anti-CD31 (550274, BD Pharmingen, San Jose, CA), and rabbit anti-NG2 chondroitin sulfate proteoglycan (AB5320, Millipore, Billerica, MA). The secondary antibodies included fluorescein-conjugated goat anti-rat IgG (ZF-0315, ZSGB-Bio, Beijing, China) and rhodamine-conjugated goat anti-rabbit IgG (ZF-0316, ZSGB-Bio, Beijing, China). Finally, the flat mount or sections were examined under a confocal microscope (TE2000-U; Nikon, Tokyo, Japan) or epifluorescence microscope (E800; Nikon, Tokyo, Japan).

## Results

### Effect of Cl_2_MDP-LIP sub-conjunctival injection on macrophage depletion

**Figure 1:**
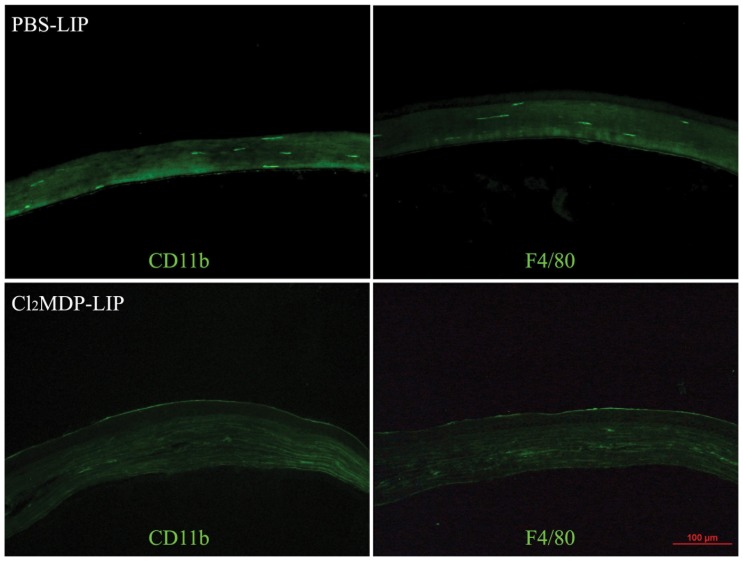
Sub-conjunctival Cl_2_MDP-LIP injection depleted corneal resident macrophages. Immunofluorescence staining of cornea after 2 days of liposome injection showed a few F4/80^+^CD11b^+^ macrophages in the central cornea of mice injected with PBS-LIP, while there was no specific staining in mice injected with clodronate liposomes.

**Figure 2:**
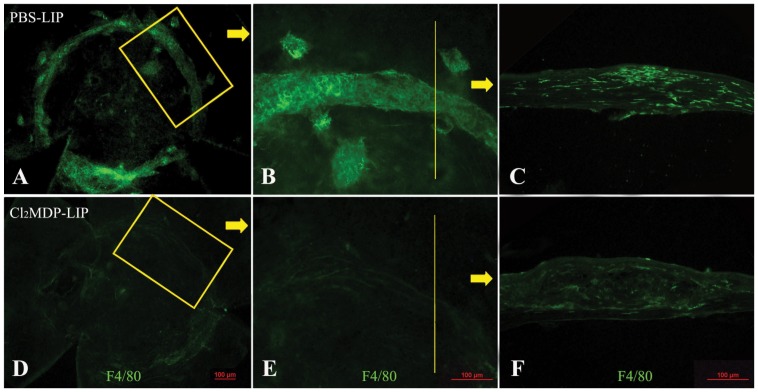
Sub-conjunctival Cl_2_MDP-LIP injection prevented the infiltration of macrophages. Corneal flat mount staining after 14 days of penetrating keratoplasty showed a number of F4/80^+^ macrophages near the suture sites and around the junction between the graft and recipient cornea (A, B). Section staining primarily revealed staining clustered in the superficial corneal stromal layer (C), while few macrophages could be found in the junction and suture area in Cl_2_MDP-LIP-injected mice (D–F).

CD11b and F4/80 are two commonly used markers for the detection of mouse macrophage [20], [21]. Immunofluorescence showed a few scattered F4/80^+^CD11b^+^ macrophages in the central corneas of mice injected with PBS-LIP (n = 5). However, there was no specific staining (2 days after the second clodronate liposome injection) in the corneas that were previously injected with clodronate liposomes, which suggests that the sub-conjunctival injection of Cl_2_MDP-LIP (n = 5) effectively depleted corneal resident phagocytes (Fig. 1). In addition, corneal flat mount staining after 14 days of penetrating keratoplasty showed a number of F4/80+ macrophages recruited near the suture sites and around the junction between the graft and recipient cornea (Fig. 2A, B). Section staining showed that the macrophages clustered in the superficial corneal stromal layer (Fig. 2C), while few macrophages were found in the junction and suture area in Cl_2_MDP-LIP-injected mice (Fig. 2D–F). The expression of CD11b also showed a similar distribution as F4/80 in PBS-LIP- and Cl_2_MDP-LIP-injected mice (data not shown). The results showed that Cl_2_MDP-LIP sub-conjunctival injection depleted not only corneal resident phagocytes, but also the infiltrated macrophages into cornea after autologous corneal transplantation for at least 14 days.

### Effect of macrophage depletion on myofibroblast differentiation

**Figure 3:**
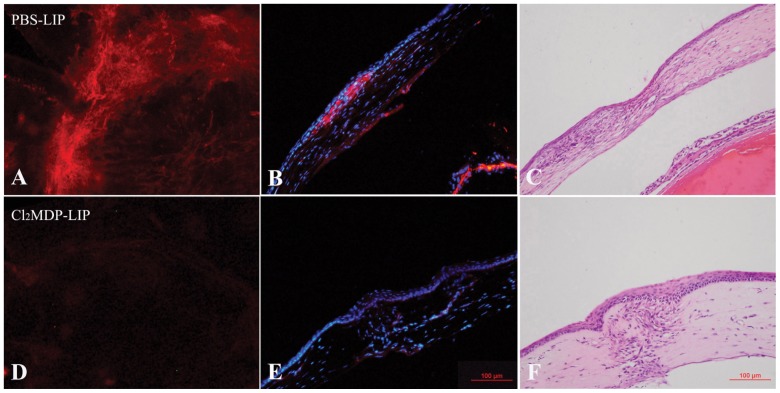
Effect of macrophage depletion on myofibroblast differentiation. Corneal flat mount staining after 14 days of penetrating keratoplasty showed that α-SMA was strongly expressed in the junction area between the graft and recipient cornea (A) and parallel to the corneal surface (B, C) in the PBS-LIP-injected mice, while in the Cl_2_MDP-LIP-treated mice, α-SMA expression was significantly lower (D), and the fibers in the junction area were more disorganized than in the control mice (E,F).

As a critical factor involved in wound contraction, myofibroblasts are characterized by α-SMA expression and are regulated by macrophages through the cytokines and chemokines released [22], [23]. Immunofluorescence staining of the corneal flat mounts after 14 days of penetrating keratoplasty revealed the strong expression of α-SMA in the junction area between the graft and the recipient cornea in the PBS-LIP-injected mice (Fig. 3A), while the mice injected with liposomes containing Cl_2_MDP showed no staining (Fig. 3D). Moreover, α-SMA staining and myofibroblast accumulation were mainly observed in the junction area and parallel to the corneal surface in the control mice (Fig. 3B,C), while in the Cl_2_MDP-LIP-treated mice, α-SMA expression was significantly lower, and the fibers in the junction area were more disorganized than in the control mice (Fig. 3E,F).

### Effect of macrophage depletion on corneal neovascularization

**Figure 4:**
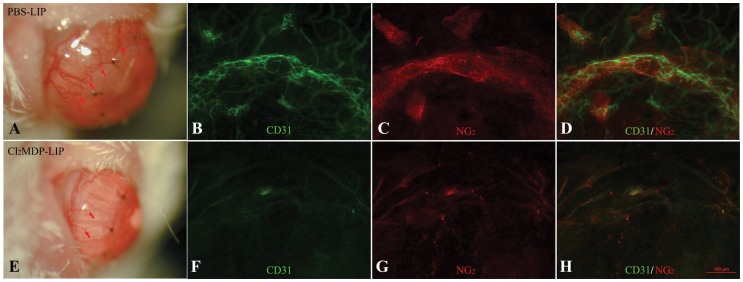
Effect of macrophage depletion on corneal neovascularization. Corneal flat mount staining after 7 days of corneal transplantation showed that both CD31 (endothelial cell marker) and NG_2_ (pericyte marker) were strongly expressed in the junction area between the graft and recipient cornea in PBS-LIP-injected mice, while the expression was significantly lower in the Cl_2_MDP-LIP-treated mice.

We evaluated the effect of macrophage depletion on corneal neovascularization caused by autologous corneal transplantation. As showed by slit microscopy observation, mice injected with PBS-LIP assumed significant vessel growth to the suture site (Fig. 4A), while the mice with depleted macrophages showed weaker neovascularization than control mice at 7 days after surgery (Fig. 4E). Immunofluorescence staining of corneal flat mounts showed CD31-positive vascular endothelial cells and NG_2_-positive pericytes in the cornea junction in PBS-treated controls (Fig. 4B–D), while there was no obvious expression of CD31 or NG_2_ in the junction of Cl_2_MDP-LIP-treated mice (Fig. 4F–H). The results suggest that the local depletion of macrophages inhibited neovascularization after autologous corneal transplantation.

### Effect of macrophage depletion and transfusion on corneal wound healing

**Figure 5:**
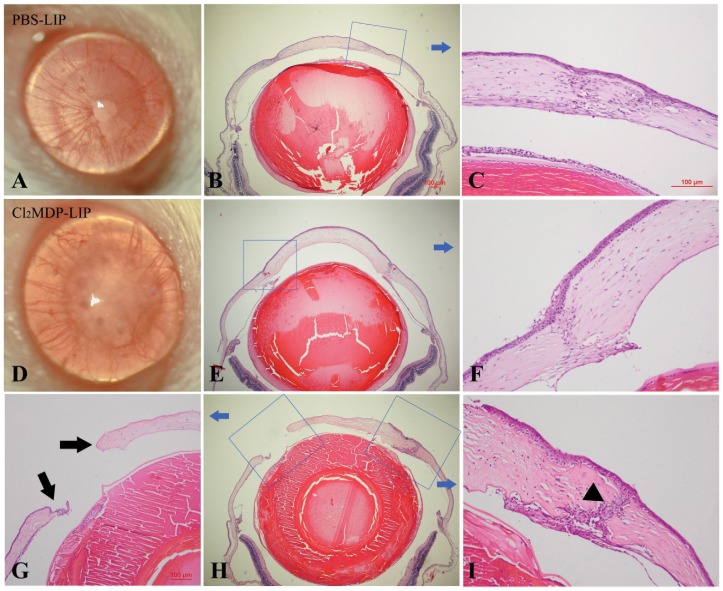
Effect of macrophage depletion on corneal wound healing. In mice injected with PBS-LIP, the graft healed well at 14 days after surgery (A), with apparent inflammatory cells and fibroblasts in the junction region between the graft and recipient cornea (B, C), and the collagen fibers were dense and distributed parallel to stromal layers (C). However, the graft showed apparent edema (D) and space between the recipient bed (E) in Cl_2_MDP-LIP-treated mice. Few inflammatory cells and fibroblasts were recruited into the junction, the collagen fibers were irregularly distributed (F), the onset of wound dehiscence was observed, with the graft detached from the recipient cornea (G, H), and there was ingrowth of corneal epithelial cells into the junction (I).

**Figure 6:**
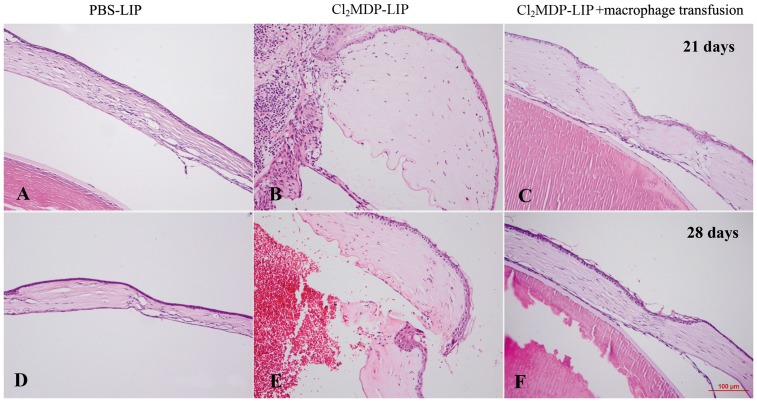
Effect of macrophage transfusion on corneal wound healing. Forty-eight hours after penetrating keratoplasty in mice injected with Cl_2_MDP-LIP, 10^5^ peritoneal macrophages from naïve Balb/c mice were transfused into conjunctiva. After 21 and 28 days later, the mice with macrophage transfusion into conjunctiva showed significant enhanced wound healing (C, F), almost similar to the control mice injected with PBS-LIP (A, D), while the macrophage-depleted mice still showed significant defective wound healing and even the disclosure of eye content (B, E).

The mouse autologous corneal transplantation model was used in the present study to exclude the interference of immune rejection caused by allogeneic transplantation. Slit-lamp photographs (100%, 25/25) and histological staining showed that the graft healed well after 14 days of penetrating keratoplasty in the PBS-LIP-treated mice (Fig. 5A). The graft was closely linked with the recipient bed, with apparent inflammatory cells and fibroblasts in the junction region between the graft and recipient cornea. The collagen fibers were dense and distributed parallel to stromal layers (Fig. 5B, C). However, the graft cornea showed apparent edema and a space between the recipient bed in the Cl_2_MDP-LIP-treated mice (64%, 16/25) (Fig. 5D). Few inflammatory cells and fibroblasts were recruited into the junction between the graft and recipient cornea. The collagen fibers were small, thin, and irregularly distributed (Fig. 5E, F). Moreover, several mice showed the onset of wound dehiscence, with the graft detached from the recipient cornea (36%, 9/25), and the ingrowth of corneal epithelial cells into the junction (Fig. 5G–I). In addition, the mice with macrophage transfusion into conjunctiva showed significant enhanced wound healing (Fig. 6C, F), almost similar to the control mice injected with PBS-LIP (Fig. 6A, D), while the macrophage-depleted mice still showed significant defective wound healing and even the disclosure of eye content (Fig. 6B, E) after 21 and 28 days of corneal transplantation.

## Discussion

Macrophages have been confirmed to play a central role during all phases of wound healing [24], [25]. During the inflammatory phase, macrophages play a role in antigen presentation, phagocytosis and the production of inflammatory cytokines and growth factors that facilitate wound healing [26], [27]. During the proliferative phase, they stimulate the proliferation of fibroblasts, endothelial cells and epithelial cells to promote ECM formation, epithelialization and neovascularization [28]. Subsequently, macrophages contribute to the remodeling of newly formed ECM by releasing degrading enzymes during the remodeling phase [29]. In the present study, for the first time, we depleted the corneal infiltrated macrophages through the sub-conjunctival injection of clodronate liposomes and evaluated the impairment of corneal wound healing after autologous corneal transplantation. The results showed that the corneal macrophage-depleted mice exhibited low inflammatory cell infiltration, irregular extracellular matrix alignment, intrastromal invasion of the corneal epithelium, and the detachment of donor corneas. Moreover, the number of macrophages, myofibroblasts, endothelial cells and pericytes was decreased in the junction area between the donor and recipient cornea. In addition, macrophages transfusion recovered the defect of corneal wound healing caused by the depletion of corneal macrophages.

The delivery of liposome containing dichloromethylene-bisphosphonate has been used popularly to deplete macrophages from liver and spleen [30], kidney [31], lung [32] and cornea [14], [33], [34] in vivo. The method is specific for the depletion of phagocytic cells, since the liposomes can be phagocytosis only by phagocytic cells. Moreover, the Cl_2_MDP cannot be easily escaped from phagocytes or enter non-phagocytic cells if leakage from liposome, and free Cl_2_MDP is not toxic by itself [35]. For cornea, several articles had described the repeated sub-conjunctival injection of Cl_2_MDP-LIP could prevent corneal allograft rejection via the depletion of infiltrated macrophages from conjunctiva in rats [33]. In the previous study, we and Van Klink also found that local depletion of macrophages may impair the immune response and aggravate fungal and acanthamoeba keratitis [14], [34]. From the depletion effect of local macrophage, Cl_2_MDP-LIP sub-conjunctival injection can deplete corneal local phagocytic cells and infiltrated macrophages for about 14 days, since we found there are only a few F4/80+ macrophages in corneal stroma after 14 days of Cl_2_MDP-LIP injection. Moreover, we also checked the cell apoptosis by TUNEL staining on the cornea of 3–21 days after penetrating keratoplasty, and found the control PBS-LIP injected mice showed more TUNEL stained cells than the Cl_2_MDP-LIP injected mice, as well as the infiltration of inflammatory cells in corneal stroma (data not shown). In the homeostatic corneas, there are residential macrophages but no residential polymorphonuclear leukocytes [36]–[40]. The results suggests the decreasing of macrophages in cornea of Cl_2_MDP-LIP injected mice after penetrating keratoplasty was caused by the depletion of resident corneal phagocytes and infiltrated macrophages from conjunctiva. It should be mentioned that neutrophils were not depleted by sub-conjunctival injection of Cl_2_MDP-LIP, since we didn't find the difference of neutrophil population in the corneal wound healing after penetrating keratoplasty (data not shown). However, although commonly used to deplete monocytes in the circulation and macrophages in tissues, Cl_2_MDP-LIP administration also depletes dendritic cells that also engulf clodronate [41]. Further study needs to confirm the specific role of macrophage and dendritic cells in corneal wound healing by using transgenic mice expressing diphtheria toxin receptors (DTR) under the promoters for either CD11b or CD11c that selectively deplete macrophage or dendritic cells [42], [43].

During stromal wound healing, fibroblasts differentiate into myofibroblasts that share similar features with smooth muscle cells, including prominent intracellular microfilament bundles and in vitro contractile responses to smooth muscle agonists [44]. Previous reports have described that the expression of α-SMA is directly correlated with corneal wound contraction, and the absence of myofibroblasts is associated with continued widening of the wound space after incisional procedures [45]. These results are similar to our results in corneal wound healing after penetrating keratoplasty. Macrophage-depleted mice showed reduced α-SMA expression, wound dehiscence and the intrastromal invasion of corneal epithelial cells, which suggested that macrophage-depleted mice exhibited impaired myofibroblast differentiation during corneal wound healing. Among the several cytokines that regulate myofibroblast differentiation and wound contraction by corneal fibroblasts, TGF-β, which is produced by infiltrated macrophages, may play a critical role during corneal wound healing. Moreover, macrophages also produce platelet-derived growth factor (PDGF), acidic and rich in cysteine (SPARC) and insulin-like growth factor-1 (IGF-1), which are involved in the process [46]–[49]. In addition, we confirmed that the elimination of macrophages resulted in the reduction of corneal neovascularization, including the number of pericytes that enclose endothelial cells to strength newly formed vessels. The reduction of the ingrowth of new vessels from the limbal region may be associated with diminished VEGF and bFGF in the wound sites of macrophage-depleted corneas [19], [50], [51]. Moreover, the suture removal at 10 days after penetrating keratoplasty was also critical for the regression of corneal neovascularization.

In conclusion, the present results showed that macrophage-depleted mice impaired corneal wound healing after autologous corneal transplantation, which was recovered by peritoneal macrophage transfusion. However, several issues need to be explored in the further study: (1) sub-conjunctival injection of Cl_2_MDP-LIP can only deplete the infiltrated macrophages in a short period, further study should be done to confirm the effect of infiltrated macrophages on corneal wound healing by using systemic injection of Cl_2_MDP-LIP. (2) Considering the limited merit of exploring the role of macrophages in autologous corneal transplantation-mediated wound healing, dendritic cells or macrophage selectively-depleted mice and corneal scratch model should be used to confirm the role of various phagocytes (neutrophils, dendritic cells or macrophages) in corneal wound healing. (3) Although we found the peritoneal macrophage transfusion could recover defect of corneal wound healing after macrophage depletion, more study should be performed by using the purified and fluorescence-labeled macrophages (such as macrophages from GFP transgenic mice) to demonstrate the detailed mechanism.

## References

[1] Mast BA, Schultz GS (1996) Interactions of cytokines, growth factors, and proteases in acute and chronic wounds. Wound Repair Regen 4: 411–420. 17309691 10.1046/j.1524-475X.1996.40404.x

[2] Behm B, Babilas P, Landthaler M, Schreml S (2012) Cytokines, chemokines and growth factors in wound healing. J Eur Acad Dermatol Venereol 26: 812–820. 22211801 10.1111/j.1468-3083.2011.04415.x

[3] Barrientos S, Stojadinovic O, Golinko MS, Brem H, Tomic-Canic M (2008) Growth factors and cytokines in wound healing. Wound Repair Regen 16: 585–601. 19128254 10.1111/j.1524-475X.2008.00410.x

[4] KR FA (2005) Wound healing: Taylor & Francis Group.

[5] Auerbach R, Lewis R, Shinners B, Kubai L, Akhtar N (2003) Angiogenesis assays: a critical overview. Clin Chem 49: 32–40. 12507958 10.1373/49.1.32

[6] Nakao S, Hata Y, Miura M, Noda K, Kimura YN, et al (2007) Dexamethasone inhibits interleukin-1beta-induced corneal neovascularization: role of nuclear factor-kappaB-activated stromal cells in inflammatory angiogenesis. Am J Pathol 171: 1058–1065. 17690185 10.2353/ajpath.2007.070172 PMC1959485

[7] Karamichos D, Guo XQ, Hutcheon AE, Zieske JD (2010) Human corneal fibrosis: an in vitro model. Invest Ophthalmol Vis Sci 51: 1382–1388. 19875671 10.1167/iovs.09-3860 PMC2868432

[8] West-Mays JA, Dwivedi DJ (2006) The keratocyte: corneal stromal cell with variable repair phenotypes. Int J Biochem Cell Biol 38: 1625–1631. 16675284 10.1016/j.biocel.2006.03.010 PMC2505273

[9] Bikbova G, Oshitari T, Tawada A, Yamamoto S (2012) Corneal changes in diabetes mellitus. Curr Diabetes Rev 8: 294–302. 22587515 10.2174/157339912800840479

[10] Lu P, Li L, Liu G, van Rooijen N, Mukaida N, et al (2009) Opposite roles of CCR2 and CX3CR1 macrophages in alkali-induced corneal neovascularization. Cornea 28: 562–569. 19421039 10.1097/ICO.0b013e3181930bcd

[11] Slegers TP, Torres PF, Broersma L, van Rooijen N, van Rij G, et al (2000) Effect of macrophage depletion on immune effector mechanisms during corneal allograft rejection in rats. Invest Ophthalmol Vis Sci 41: 2239–2247. 10892868

[12] Slegers TP, van der Gaag R, van Rooijen N, van Rij G, Streilein JW (2003) Effect of local macrophage depletion on cellular immunity and tolerance evoked by corneal allografts. Curr Eye Res 26: 73–79. 12815525 10.1076/ceyr.26.2.73.14510

[13] Van der Veen G, Broersma L, Dijkstra CD, Van Rooijen N, Van Rij G, et al (1994) Prevention of corneal allograft rejection in rats treated with subconjunctival injections of liposomes containing dichloromethylene diphosphonate. Invest Ophthalmol Vis Sci 35: 3505–3515. 8056526

[14] Hu J, Wang Y, Xie L (2009) Potential role of macrophages in experimental keratomycosis. Invest Ophthalmol Vis Sci 50: 2087–2094. 19074808 10.1167/iovs.07-1237

[15] Hunter MM, Wang A, Parhar KS, Johnston MJ, Van Rooijen N, et al (2010) In vitro-derived alternatively activated macrophages reduce colonic inflammation in mice. Gastroenterology 138: 1395–1405. 20044996 10.1053/j.gastro.2009.12.041

[16] Chen L, Huq S, Gardner H, de Fougerolles AR, Barabino S, et al (2007) Very late antigen 1 blockade markedly promotes survival of corneal allografts. Arch Ophthalmol 125: 783–788. 17562989 10.1001/archopht.125.6.783 PMC2677688

[17] Cunnusamy K, Chen PW, Niederkorn JY (2011) IL-17A-dependent CD4+CD25+ regulatory T cells promote immune privilege of corneal allografts. J Immunol 186: 6737–6745. 21551366 10.4049/jimmunol.1100101 PMC3110606

[18] Liu Y, Hamrah P, Zhang Q, Taylor AW, Dana MR (2002) Draining lymph nodes of corneal transplant hosts exhibit evidence for donor major histocompatibility complex (MHC) class II-positive dendritic cells derived from MHC class II-negative grafts. J Exp Med 195: 259–268. 11805152 10.1084/jem.20010838 PMC2193609

[19] Cursiefen C, Chen L, Borges LP, Jackson D, Cao J, et al (2004) VEGF-A stimulates lymphangiogenesis and hemangiogenesis in inflammatory neovascularization via macrophage recruitment. J Clin Invest 113: 1040–1050. 15057311 10.1172/JCI20465 PMC379325

[20] Schiechl G, Bauer B, Fuss I, Lang SA, Moser C, et al (2011) Tumor development in murine ulcerative colitis depends on MyD88 signaling of colonic F4/80+CD11b(high)Gr1(low) macrophages. J Clin Invest 121: 1692–1708. 21519141 10.1172/JCI42540 PMC3083803

[21] Hussain S, Stohlman SA (2012) Peritoneal macrophage from male and female SJL mice differ in IL-10 expression and macrophage maturation. J Leukoc Biol 91: 571–579. 22262797 10.1189/jlb.0711351 PMC3996205

[22] Barron L, Wynn TA (2011) Fibrosis is regulated by Th2 and Th17 responses and by dynamic interactions between fibroblasts and macrophages. Am J Physiol Gastrointest Liver Physiol 300: G723–728. 21292997 10.1152/ajpgi.00414.2010 PMC3302189

[23] Murray LA, Chen Q, Kramer MS, Hesson DP, Argentieri RL, et al (2011) TGF-beta driven lung fibrosis is macrophage dependent and blocked by Serum amyloid P. Int J Biochem Cell Biol 43: 154–162. 21044893 10.1016/j.biocel.2010.10.013

[24] Mahdavian Delavary B, van der Veer WM, van Egmond M, Niessen FB, Beelen RH (2011) Macrophages in skin injury and repair. Immunobiology 216: 753–762. 21281986 10.1016/j.imbio.2011.01.001

[25] Lucas T, Waisman A, Ranjan R, Roes J, Krieg T, et al (2010) Differential roles of macrophages in diverse phases of skin repair. J Immunol 184: 3964–3977. 20176743 10.4049/jimmunol.0903356

[26] Baum CL, Arpey CJ (2005) Normal cutaneous wound healing: clinical correlation with cellular and molecular events. Dermatol Surg 31: 674–686; discussion 686. 15996419 10.1111/j.1524-4725.2005.31612

[27] Khallou-Laschet J, Varthaman A, Fornasa G, Compain C, Gaston AT, et al (2010) Macrophage plasticity in experimental atherosclerosis. PLoS One 5: e8852. 20111605 10.1371/journal.pone.0008852 PMC2810335

[28] Porcheray F, Viaud S, Rimaniol AC, Leone C, Samah B, et al (2005) Macrophage activation switching: an asset for the resolution of inflammation. Clin Exp Immunol 142: 481–489. 16297160 10.1111/j.1365-2249.2005.02934.x PMC1809537

[29] Koh TJ, DiPietro LA (2011) Inflammation and wound healing: the role of the macrophage. Expert Rev Mol Med 13: e23. 21740602 10.1017/S1462399411001943 PMC3596046

[30] Kurahashi K, Sawa T, Ota M, Kajikawa O, Hong K, et al (2009) Depletion of phagocytes in the reticuloendothelial system causes increased inflammation and mortality in rabbits with Pseudomonas aeruginosa pneumonia. Am J Physiol Lung Cell Mol Physiol 296: L198–209. 19028978 10.1152/ajplung.90472.2008 PMC2643994

[31] Jang HS, Kim J, Park YK, Park KM (2008) Infiltrated macrophages contribute to recovery after ischemic injury but not to ischemic preconditioning in kidneys. Transplantation 85: 447–455. 18301336 10.1097/TP.0b013e318160f0d1

[32] Forbes A, Pickell M, Foroughian M, Yao LJ, Lewis J, et al (2007) Alveolar macrophage depletion is associated with increased surfactant pool sizes in adult rats. J Appl Physiol 103: 637–645. 17446406 10.1152/japplphysiol.00995.2006

[33] Slegers TP, Broersma L, van Rooijen N, Hooymans JM, van Rij G, et al (2004) Macrophages play a role in the early phase of corneal allograft rejection in rats. Transplantation 77: 1641–1646. 15201661 10.1097/01.tp.0000129410.89410.f2

[34] van Klink F, Taylor WM, Alizadeh H, Jager MJ, van Rooijen N, et al (1996) The role of macrophages in Acanthamoeba keratitis. Invest Ophthalmol Vis Sci 37: 1271–1281. 8641830

[35] Van Rooijen N, Sanders A (1994) Liposome mediated depletion of macrophages: mechanism of action, preparation of liposomes and applications. J Immunol Methods 174: 83–93. 8083541 10.1016/0022-1759(94)90012-4

[36] Chinnery HR, Humphries T, Clare A, Dixon AE, Howes K, et al (2008) Turnover of bone marrow-derived cells in the irradiated mouse cornea. Immunology 125: 541–548. 18540963 10.1111/j.1365-2567.2008.02868.x PMC2612551

[37] Xu H, Chen M, Reid DM, Forrester JV (2007) LYVE-1-positive macrophages are present in normal murine eyes. Invest Ophthalmol Vis Sci 48: 2162–2171. 17460275 10.1167/iovs.06-0783

[38] Lin M, Carlson E, Diaconu E, Pearlman E (2007) CXCL1/KC and CXCL5/LIX are selectively produced by corneal fibroblasts and mediate neutrophil infiltration to the corneal stroma in LPS keratitis. J Leukoc Biol 81: 786–792. 17110418 10.1189/jlb.0806502 PMC3909486

[39] Carlson EC, Lin M, Liu CY, Kao WW, Perez VL, et al (2007) Keratocan and lumican regulate neutrophil infiltration and corneal clarity in lipopolysaccharide-induced keratitis by direct interaction with CXCL1. J Biol Chem 282: 35502–35509. 17911102 10.1074/jbc.M705823200 PMC3909483

[40] Carlson EC, Drazba J, Yang X, Perez VL (2006) Visualization and characterization of inflammatory cell recruitment and migration through the corneal stroma in endotoxin-induced keratitis. Invest Ophthalmol Vis Sci 47: 241–248. 16384969 10.1167/iovs.04-0741

[41] Lu L, Faubel S, He Z, Andres Hernando A, Jani A, et al (2012) Depletion of macrophages and dendritic cells in ischemic acute kidney injury. Am J Nephrol 35: 181–190. 22286667 10.1159/000335582 PMC3326279

[42] Probst HC, Tschannen K, Odermatt B, Schwendener R, Zinkernagel RM, et al (2005) Histological analysis of CD11c-DTR/GFP mice after in vivo depletion of dendritic cells. Clin Exp Immunol 141: 398–404. 16045728 10.1111/j.1365-2249.2005.02868.x PMC1809468

[43] Qi F, Adair A, Ferenbach D, Vass DG, Mylonas KJ, et al (2008) Depletion of cells of monocyte lineage prevents loss of renal microvasculature in murine kidney transplantation. Transplantation 86: 1267–1274. 19005409 10.1097/TP.0b013e318188d433

[44] Majno G, Gabbiani G, Hirschel BJ, Ryan GB, Statkov PR (1971) Contraction of granulation tissue in vitro: similarity to smooth muscle. Science 173: 548–550. 4327529 10.1126/science.173.3996.548

[45] Jester JV, Petroll WM, Barry PA, Cavanagh HD (1995) Expression of alpha-smooth muscle (alpha-SM) actin during corneal stromal wound healing. Invest Ophthalmol Vis Sci 36: 809–819. 7706029

[46] Daniels JT, Khaw PT (2000) Temporal stimulation of corneal fibroblast wound healing activity by differentiating epithelium in vitro. Invest Ophthalmol Vis Sci 41: 3754–3762. 11053273

[47] Kurosaka H, Kurosaka D, Kato K, Mashima Y, Tanaka Y (1998) Transforming growth factor-beta 1 promotes contraction of collagen gel by bovine corneal fibroblasts through differentiation of myofibroblasts. Invest Ophthalmol Vis Sci 39: 699–704. 9538875

[48] Werner S, Grose R (2003) Regulation of wound healing by growth factors and cytokines. Physiol Rev 83: 835–870. 12843410 10.1152/physrev.2003.83.3.835

[49] Ishida Y, Gao JL, Murphy PM (2008) Chemokine receptor CX3CR1 mediates skin wound healing by promoting macrophage and fibroblast accumulation and function. J Immunol 180: 569–579. 18097059 10.4049/jimmunol.180.1.569

[50] Jin Y, Arita M, Zhang Q, Saban DR, Chauhan SK, et al (2009) Anti-angiogenesis effect of the novel anti-inflammatory and pro-resolving lipid mediators. Invest Ophthalmol Vis Sci 50: 4743–4752. 19407006 10.1167/iovs.08-2462 PMC2763387

[51] Chung ES, Chauhan SK, Jin Y, Nakao S, Hafezi-Moghadam A, et al (2009) Contribution of macrophages to angiogenesis induced by vascular endothelial growth factor receptor-3-specific ligands. Am J Pathol 175: 1984–1992. 19808642 10.2353/ajpath.2009.080515 PMC2774062

